# Mineralogical and Geochemical Fingerprinting of Potentially Toxic Elements (PTEs) in Asbestos and Non‐Asbestos Tremolite: Implications for Human Health

**DOI:** 10.1029/2026GH001853

**Published:** 2026-05-20

**Authors:** A. Bloise, I. Fuoco, G. Vespasiano, E. Giorno, A. Pacella, S. Filicetti, M. F. La Russa, D. Pereira, C. Piersante, C. Apollaro

**Affiliations:** ^1^ Department of Biology, Ecology and Earth Sciences University of Calabria Rende Italy; ^2^ University Museum System—SiMU University of Calabria Rende Italy; ^3^ Research Group CHARROCK University of Salamanca Salamanca Spain; ^4^ Department of Chemistry and Chemical Technology Department University of Calabria Rende Italy; ^5^ Department of Earth Sciences Sapienza University of Rome Rome Italy; ^6^ Geology Department, Science Faculty University of Salamanca Salamanca Spain

**Keywords:** asbestos tremolite, non‐asbestos tremolite, potentially toxic elements

## Abstract

This paper aims to comprehensively investigate the content of potentially toxic elements (PTEs) in 11 tremolite samples to better understanding of their potential effects on human health. Accurate characterization of trace element concentrations in asbestos mineral fibers is crucial to elucidate their potential synergistic contribution to the mechanisms of asbestos‐induced carcinogenesis and related pathologies, particularly in light of the documented involvement of elements such as Ni and Cr in the etiology of lung cancer. Samples were collected from diverse geological settings: San Severino Lucano and Iacolinei (Basilicata region, South Italy), Val Malenco (Lombardy region, North Italy), Praborna and Verrayes (Aosta Valley, North Italy), Monastero di Lanzo, Bracchiello, Caprie (Piedmont region, North Italy), Reventino (Calabria region, South Italy), Campolungo (Ticino Alps, Swiss), and Fowler (St. Lawrence Co., New York, USA). PTEs concentrations were determined using Inductively Coupled Plasma Optical Emission Spectrometry. The distribution of PTEs among different tremolite types was compared and discussed to provide a comprehensive overview of the data set.Tremolite asbestos samples showed variable concentrations of trace elements, with Mn (691.5 ppm) and Ni (474.2 ppm) being the most abundant. Samples from Monastero di Lanzo exhibited the highest total PTEs content (4,709 ppm). Statistical analyses revealed a consistent geochemical contrast: asbestos tremolite is systematically enriched in Mn and Ni, leading to higher overall PTEs levels, while prismatic tremolite is defined by very low Mn–Ni contents. The observed elemental variability reflects distinct geological settings that influence PTEs incorporation and potentially affect toxicity.

## Plain Language Summary

Tremolite asbestos is a known cause of severe diseases such as lung cancer. In this study, we investigated whether the concentration of potentially toxic elements (PTEs) differs between asbestos and non‐asbestos (prismatic) tremolite, which could contribute to their differing pathogenic effects. We analyzed 11 tremolite samples collected from different geological settings in Italy, Switzerland, and the United States. Using laboratory‐based analytical techniques, we quantified PTEs (e.g., Ni, Cr, Mn) due to their established role in carcinogenesis. Our results revealed a clear and consistent geochemical distinction: asbestos tremolite contains significantly higher concentrations of these PTEs, whereas non‐asbestos (prismatic) tremolite shows very low levels. These findings suggest that the toxicity of asbestos tremolite may be enhanced not only by its fibrous morphology but also by its distinct chemical composition. Understanding this difference is important for better assessing health risks related to natural asbestos exposure and for improving prevention and regulatory strategies.

## Key Points

- Potentially toxic elements (PTEs) were determined for various tremolite asbestos by Inductively Coupled Plasma Optical Emission Spectrometry
- Mn and Ni are the predominant PTEs in tremolite
- Crystal morphology critically controls PTEs incorporation

## 1 Introduction

The commercial term “asbestos” refers to six fibrous minerals classified into two main groups: serpentine (chrysotile) and amphiboles (asbestos tremolite, asbestos actinolite, asbestos anthophyllite, amosite, crocidolite) (WHO, 1986). Despite the ban on the use and commercialization of asbestos in many countries, environmental exposure remains a significant concern (Gualtieri, 2017). Tremolite crystals are commonly found in various geological environments (Belluso et al., 2017; Bloise et al., 2014, 2017, 2020) and their disturbance can release breathable fibers (length >5 μm, aspect ratio ≥3:1) (Gualtieri, 2017; Pacella et al., 2024). Owing to its favorable technological properties, tremolite was historically mined in the United States at sites including the Centreville quarry (Virginia) and the Teeter quarry (Pennsylvania) (Addison & McConnell, 2008; Bernstein, 1980; De Luca et al., 2013; Geyer et al., 1976; Medici, 1972; Van Gosen et al., 2004). Tremolite has been used in asbestos‐containing materials, although less extensively than other regulated amphiboles such as crocidolite and amosite (Bloise, Catalano, & Gualtieri, 2018; Bloise, Kusiorowski, & Gualtieri, 2018; Gualtieri, 2012; Gualtieri, Gandolfi, et al., 2018). It often occurs as an impurity in widely used minerals such as talc, vermiculite, and chrysotile asbestos (Kleinfeld et al., 1974; Ross et al., 1968; Van Gosen et al., 2004; Virta, 1989), which are commercial applied in cement, cosmetics, pharmaceutical products, paint, and other materials (Gaffney et al., 2017; Huuskonen et al., 1980). For example, the use of talcum baby powder contaminated with asbestos tremolite has been linked to the development of cancer in consumers (Carbone et al., 2013; Kazan‐Allen, 2019; Moline et al., 2020). It has been widely documented that tremolite separated from chrysotile asbestos was used to plaster the walls of buildings throughout the Middle East a practice that has resulted in numerous deaths (Kogel et al., 2006). The IARC classifies tremolite asbestos as a Group 1 carcinogen (IARC, 2012) because inhalation can cause a range of respiratory diseases when inhaled (Addison & McConnell, 2008; Donaldson et al., 2010; Lippmann, 2014). The toxicity and pathogenicity of asbestos tremolite result from the synergistic interplay of several factors, including morphology, fiber size, surface reactivity, and biopersistence (Berry et al., 2022; Gualtieri, 2018). The presence of potentially toxic elements (PTEs, i.e., Fe, Cr, Ni, Mn, Co, Pb) within the structure of asbestos fibers, in combination with the other parameters listed above, results as an additional concern in the context of asbestos toxicity (Bloise et al., 2016, 2020; Cralley et al., 1968; Dixon et al., 1970; Gualtieri, Pollastri, et al., 2018). When inhaled, asbestos fibers may, after a latency period, dissolve within the lungs, releasing their toxic cargo and potentially causing cellular damage (Cairns et al., 2023; Nemery, 1990; Wei et al., 2014). Indeed, substantial evidence has demonstrated that toxic elements, even at low concentrations, induce significant cellular oxidative stress by increasing the generation of reactive oxygen species (ROS) and, in some cases, by causing DNA disruptions through direct binding (Caicedo et al., 2007; Chen et al., 2013; IARC, 2012; Scharf et al., 2014).

For example, high concentrations of Cr and Co in the human body, potentially from asbestos exposure, can lead to oxidative protein damage and a consequent loss of function (Scharf et al., 2014). Concerning Ni, its toxicity depends on the route of exposure, but studies have shown that the accumulation of this element in the lungs represents the most significant health risk (Sutherland & Costa, 2002). Due to its high capacity to generate ROS, Ni is also known to induce pulmonary fibrosis and respiratory tract cancer (Bai et al., 2018; Oller et al., 1997; Pan et al., 2018; Seilkop & Oller, 2003). IARC has classified both soluble and insoluble Ni compounds as Group 1 carcinogens (Nackerdien et al., 1991). In vitro studies have shown that Ni in asbestos fibers of various sizes induces cell death through apoptosis and necrosis after 24 hr of exposure (Perez et al., 2016). Additionally, inhalation of asbestos‐containing dust has been correlated with carcinogenesis in workers exposed to Ni processing (Perez et al., 2016). Notably, in aquatic environments with elevated concentrations of chrysotile asbestos in sediment, fish species have exhibited high levels of Ni and Mn in their muscle tissues, along with other growth‐related anomalies (Guagliardi et al., 2016; Schreier et al., 1987). Similarly, rats exposed to asbestos dust containing high amounts of Cr, Ni, and Co showed a high incidence of lung cancer, as reported by Gross et al. (1969).

Zn is an essential trace element, involved in numerous enzymatic reactions and fundamental biological processes (Jomova et al., 2022; Rubio et al., 2007). However, excessive exposure to zinc compounds can cause irritation of the respiratory tract, with acute symptoms such as coughing, shortness of breath, and chest pain, affecting the mucous membranes, pharynx, and the entire respiratory system (ATSDR, 2005; Wu et al., 2013). This dual role of zinc, essential yet potentially toxic, highlights the need to assess not only its presence but also its concentration within asbestos fibers. Several studies have reported associations between Mn exposure and adverse health effects (Hernández‐Pellón & Fernández‐Olmo, 2019; Myeong et al., 2009; Riojas‐Rodríguez et al., 2010; Zoni et al., 2007). Accumulation of Mn in the human body can lead to various dysfunctions, including neurofunctional changes and ultimately lethal diseases. Prolonged exposure to low levels of Mn can significantly affect the central nervous system.

Regarding lead, 50% of Pb deposited in the respiratory tract is absorbed into the systemic circulation (Levin & Goldberg, 2000). Many authors agree on the inflammatory role of Pb on cells, as it can mimic the functions of other bivalent ions like Ca, Mg, and Fe. This disruption leads to disturbances in cellular homeostasis and alters various biological processes such as protein folding, apoptosis, ion transport, and oxidant‐antioxidant balance (Boskabady et al., 2018; Farkhondeh et al., 2017; Jacobs et al., 2009). In this context, it is not surprising that some authors describe asbestos as a carrier for PTEs (Bloise et al., 2016; Bowes & Farrow, 1997; Dixon et al., 1970; Nemery, 1990). Accordingly, assessing PTEs content in asbestos tremolite can provide insights into its pathogenicity and potential impact on human health. Within this framework, the present study aims to quantify and compare the concentrations of PTEs (Ag, As, Ba, Co, Cr, Cu, Li, Mn, Ni, Pb, Sn, Zn, Zr) in 11 tremolite samples analyzed by using ICP‐OES. These data are essential not only for advancing research on the pathogenicity and health impact of asbestos fibers but also for developing chemical criteria to discriminate between asbestos and non‐asbestos tremolite varieties.

## 2 Materials and Methods

Table 1 reports the 11 tremolite samples investigated in this study, including tremolite asbestos from San Severino Lucano (Basilicata region, South Italy), Val Malenco (Lombardy region, North Italy), Praborna (Aosta Valley region, North Italy), Monastero di Lanzo (Piedmont Region, North Italy), Iacolinei (Basilicata region, South Italy), Reventino (Calabria region, South Italy),Verrayes (Aosta Valley region, North Italy), Bracchiello (Piedmont region North Italy), and tremolite not‐asbestos from Fowler (St. Lawrence Co., New York, USA), Caprie (Piedmont region North Italy) and Campolungo (Ticino Alps, Swiss).

**Table 1:** Investigated Asbestos Tremolite Samples and Main Characteristics

Sample	Provenance	^a^Mineralogical composition	Morphology	Mineral species
Tr. San Severino Lucano	Basilicata region (South Italy)	Trem >> Serp	Fibrous	Asbestos
Tr. Val Malenco	Lombardy region (North Italy)	Trem	Fibrous	Asbestos
Tr. Praborna	Aosta Valley region (North Italy)	Trem >> Serp	Fibrous	Asbestos
Tr. Monastero di Lanzo	Piedmont region (North Italy)	Trem >> Serp	Fibrous	Asbestos
Tr. Bracchiello	Piedmont region (North Italy)	Trem	Fibrous	Asbestos
Tr. Reventino	Calabria region (South Italy)	Trem >> Serp	Fibrous	Asbestos
Tr. Verrayes	Aosta Valley region (North Italy)	Trem >> Talc > Serp	Fibrous	Asbestos
Tr. Fowler	St. Lawrence Co. (New York, USA)	Talc > Trem	Prismatic	Not‐asbestos
Tr. Campolungo	Ticino Alps (Swiss)	Trem >> Talc > Dol > Cal	Prismatic	Not‐asbestos
Tr. Caprie	Piedmont region (North Italy)	Trem >> Talc	Prismatic	Not‐asbestos
Tr. Iacolinei	Basilicata region (South Italy)	Trem >> Serp	Fibrous	Asbestos

^a^ Mineralogical composition data from Bloise, 2023.

Each sample was carefully examined under a binocular microscopy to ensure the fibers were free from contamination by other mineral impurities, which are typically present in small amounts in the raw material (Table 1). Although impurities were manually removed, the procedure could not fully eliminate micro‐ and nano‐sized particles. Therefore, their potential contribution to the chemical composition remains cannot be ruled out. The selected fibers were then powdered using an agate mortar. The chemical data for the major elements are provided by Bloise (2023) (Table S1 in Supporting Information S1), whereas the trace element concentrations were detected in this work.

### 2.1 ICP‐OES

Trace element concentrations (Ag, As, Ba, Co, Cr, Cu, Li, Mn, Ni, Pb, Sn, Zn, Zr) were determined using Inductively Coupled Plasma Optical Emission Spectroscopy (ICP‐OES), employing an Agilent 5800 VDV system. In the first step approximately 100 mg of each powdered sample was dissolved in a mixture of Merck “Suprapur” grade hydrofluoric acid (6 mL HF) and nitric acid (4 mL HNO_3_). Sample digestion was performed using a Milestone ETHOS UP microwave digestion system equipped with HPR‐1,000/10 high‐pressure TFM vessels. After complete dissolution, a small amount of high‐purity boric acid (H_3_BO_3_) was added to complex any residual fluoride ions and to stabilize the matrix for subsequent ICP‐OES analysis. This step is critical to prevent damage to the torch and cones and to minimize signal suppression caused by fluoride complexes. Calibration curves were constructed using O_2_Si “Multielement Smart Solutions,” covering all elements analyzed. Instrumental limits of quantification (LOQs) were evaluated using the “white method” and are consistent with the ISO 11885 standard: water quality determination of selected elements by ICP‐OES. Concentrations below the LOQ were reported as “below detection limit” (b.d.l.). To ensure analytical reproducibility and precision, all measurements were conducted in triplicate. Instrumental drift and matrix effects were monitored throughout the analytical run using quality control solutions and periodic re‐analysis of standards.

### 2.2 Statistical Analysis

To evaluate the internal variability of the data set and the compositional differences between fibrous and prismatic tremolite, a univariate and multivariate statistical analysis was performed using PAST 4.17. The analysis was performed on a range of elements, with the focus being on Cr, Cu, Mn, and Ni, given their presence in all samples and significant variability. For each of these, the primary descriptive parameters were calculated (mean, range, standard deviation, coefficient of variation, quartiles, skewness, and kurtosis). The differences between the two morphologies were verified using a two‐sample *t*‐test, while the multivariate distinction was evaluated using MANOVA. The data structure was explored using Principal Component Analysis (PCA) on standardized data (z‐score), and the main discriminating variable between fibrous and prismatic tremolite was identified using Linear Discriminant Analysis (LDA). Finally, the similarity relationships between samples were analyzed using Hierarchical Cluster Analysis (HCA) with the UPGMA aggregation method and the Euclidean distance. The employment of a two‐way clustering approach facilitated the concurrent generation of the dendrograms of the samples and variables, accompanied by the relative heatmap.

## 3 Results

**Figure 1:**
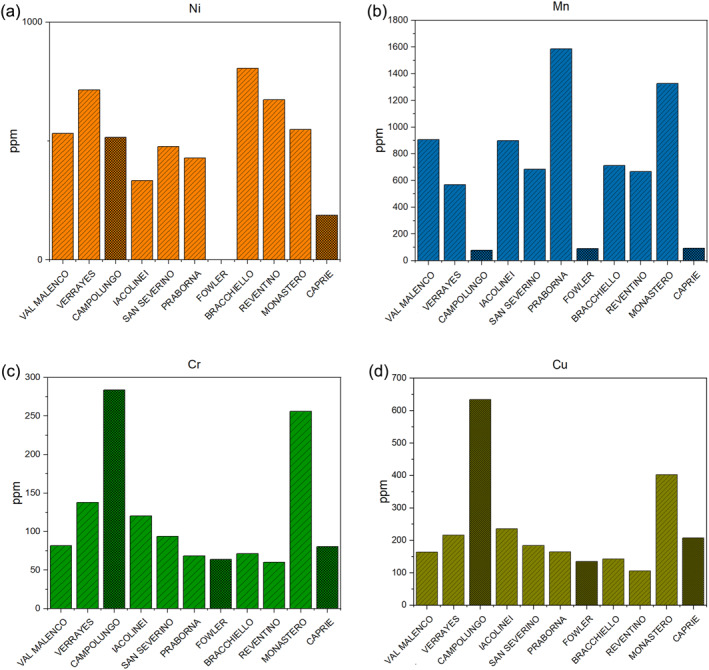
Concentrations of Cr, Ni, Cu, and Mn (ppm) detected in the 11 tremolite samples: San Severino Lucano, Val Malenco, Praborna, Monastero di Lanzo, Bracchiello, Reventino, Verrayes, Fowler, Campolungo, Caprie, Iacolinei analyzed by Inductively Coupled Plasma Optical Emission Spectrometry. The dotted and dark columns refer to non‐asbestos tremolite samples (Fowler, Campolungo, Caprie).

The investigated tremolite samples shown considerable compositional variability across the sites, with some samples displaying particularly high concentrations of specific elements (Figure 1; Table 2 and Figure S1 in Supporting Information S1).

**Table 2:** Trace Element Contents (ppm) in the Investigated Samples by Inductively Coupled Plasma Optical Emission Spectrometry

	Ag	As	Ba	Co	Cr	Cu	Li	Mn	Ni	Pb	Sn	Zn	Zr
Tr. Val Malenco	b.d.l.	b.d.l.	b.d.l.	b.d.l.	81.87 ± 0.01	163.74 ± 0.01	b.d.l.	906.43 ± 0.01	532.16 ± 0.01	b.d.l.	b.d.l.	110.53 ± 0.01	57.31 ± 0.01
Tr. Verrayes	b.d.l.	b.d.l.	b.d.l.	b.d.l.	137.93 ± 0.01	215.52 ± 0.01	b.d.l.	568.97 ± 0.01	715.52 ± 0.03	b.d.l.	b.d.l.	108.62 ± 0.01	b.d.l.
Tr. Campolungo	b.d.l.	b.d.l.	b.d.l.	b.d.l.	283.69 ± 0.01	633.57 ± 0.01	b.d.l.	78.01 ± 0.01	515.36 ± 0.01	b.d.l.	b.d.l.	347.52 ± 0.04	b.d.l.
Tr. Fowler	b.d.l.	b.d.l.	b.d.l.	b.d.l.	63.87 ± 0.01	134.55 ± 0.01	14.66 ± 0.01	88.48 ± 0.01	b.d.l.	b.d.l.	b.d.l.	68.06 ± 0.01	b.d.l.
Tr. Caprie	b.d.l.	b.d.l.	b.d.l.	b.d.l.	80.62 ± 0.01	207.67 ± 0.01	b.d.l.	93.46 ± 0.01	187.34 ± 0.01	b.d.l.	b.d.l.	206.74 ± 0.01	b.d.l.
Tr. Iacolinei	b.d.l.	b.d.l.	b.d.l.	b.d.l.	120.37 ± 0.01	235.19 ± 0.01	b.d.l.	898.15 ± 0.01	333.33 ± 0.01	b.d.l.	b.d.l.	119.44 ± 0.01	b.d.l.
Tr. San Severino Lucano	b.d.l.	b.d.l.	b.d.l.	b.d.l.	93.66 ± 0.01	183.69 ± 0.01	b.d.l.	684.59 ± 0.01	476.74 ± 0.01	b.d.l.	b.d.l.	88.04 ± 0.01	b.d.l.
Tr. Praborna	b.d.l.	66.96 ± 0.01	b.d.l.	208.56 ± 0.01	68.61 ± 0.01	164.65 ± 0.01	b.d.l.	1,586.17 ± 0.01	428.10 ± 0.01	b.d.l.	b.d.l.	195.39 ± 0.04	b.d.l.
Tr. Bracchiello	b.d.l.	b.d.l.	b.d.l.	b.d.l.	71.15 ± 0.01	142.29 ± 0.01	b.d.l.	711.46 ± 0.01	805.34 ± 0.01	b.d.l.	b.d.l.	b.d.l.	b.d.l.
Tr. Reventino	b.d.l.	b.d.l.	b.d.l.	b.d.l.	60.24 ± 0.01	105.31 ± 0.01	b.d.l.	665.54 ± 0.01	673.97 ± 0.01	b.d.l.	b.d.l.	68.24 ± 0.02	35.89 ± 0.03
Tr. Monastero di Lanzo	20.11 ± 0.01	b.d.l.	89.58 ± 0.01	b.d.l.	255.94 ± 0.01	402.19 ± 0.01	648.99 ± 0.01	1,325.41 ± 0.01	548.45 ± 0.01	18.28 ± 0.01	26.50 ± 0.01	136.74 ± 0.02	22.88 ± 0.01

*Note.* b.d.l. = below detection limit.

The tremolite sample from Bracchiello, in Piedmont region (North Italy), exhibited the highest Ni concentration (805.34 ppm). In contrast, the tremolite from Fowler (St. Lawrence County, New York, USA) had the lowest concentration, which was below detection limit (b.d.l.) (Figure 1a). The highest concentration of Mn (1,586.17 ppm) was found in the tremolite from Praborna, located in the Valle d'Aosta region of Northern Italy, while the lowest concentration (78.01 ppm) was observed in the Campolungo tremolite (Figure 1b). For Cr and Cu, the highest concentrations were found in the Campolungo tremolite, with values of 283.69 ppm for Cr (Figure 1c) and 633.57 ppm for Cu (Figure 1d). Conversely, the lowest concentrations of these elements were detected in the Reventino tremolite from Calabria, Southern Italy, with values of 60.24 ppm for Cr and 105.31 ppm for Cu (Figure 1d). The highest concentration of Zn was observed in the tremolite from Campolungo (347.52 ppm), whereas the lowest concentration was found in the tremolite from Bracchiello, Piedmont, Northern Italy, which was b.d.l. (Figure S2 in Supporting Information S1). Except for one sample, all values for silver (Ag), arsenic (As), barium (Ba), cobalt (Co), lead (Pb), and tin (Sn) were below the detection limit (b.d.l.). The values obtained in the tremolite from Monastero di Lanzo, Piedmont, Northern Italy, were Ag (20.11 ppm), Pb (18.28 ppm), and Sn (26.50 ppm). As and Co were only found in the Praborna tremolite, with concentrations of 66.96 and 208.56 ppm, respectively. Barium was detected exclusively in the Monastero di Lanzo tremolite, with a concentration of 89.58 ppm. Lithium was b.d.l. in all samples except for Monastero di Lanzo tremolite, which exhibited a value of 648.99 ppm, and the Fowler tremolite, with a value of 14.66 ppm. Regarding zirconium (Zr), all values were b.d.l., except for three samples: Monastero di Lanzo tremolite (22.88 ppm), the Val Malenco tremolite (57.31 ppm) from Lombardy, Northern Italy, and the Reventino tremolite (35.89 ppm) (Figure S1 in Supporting Information S1).

### 3.1 Statistical Analysis

**Figure 2:**
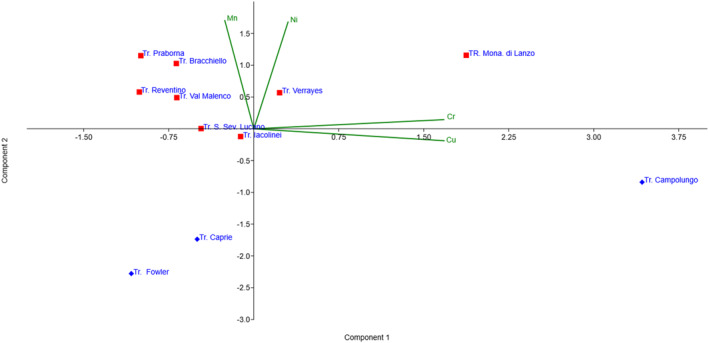
Principal component analysis biplot based on Cr, Cu, Mn, and Ni concentrations in the analyzed tremolite samples. Red squares indicate fibrous tremolite and blue rhombuses prismatic (non‐asbestiform) ones. The first two components explain 83.6% of the variance. Green vectors show element loadings, with Mn and Ni as the main contributors to the separation between the two morphologies.

The internal variability of the data set was assessed through univariate and multivariate statistical analyses. The two‐sample *t*‐test showed that only Mn displays a statistically significant difference between fibrous and prismatic tremolite (*p* = 0.00031), while Cr, Cu, and Ni do not, although Ni exhibits a non‐significant trend toward higher values in fibrous samples. Multivariate analyses further supported the distinction between the two morphologies. MANOVA indicated that the combined chemical profile of Cr, Cu, Mn, and Ni significantly separates fibrous from prismatic tremolite (*p* = 0.00335; Wilks' *λ* = 0.096). Principal Component Analysis (PCA), conducted on standardised data, showed a clear separation between the two groups along the first two components, which together explain 83.6% of the total variance (Figure 2).

**Figure 3:**
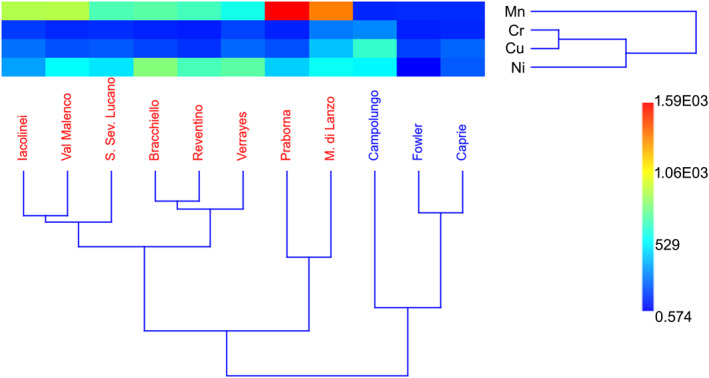
Two‐way Hierarchical Cluster Analysis with heatmap and dendrogram of tremolite samples based on Cr, Cu, Mn, and Ni (Euclidean distance, UPGMA).

Linear Discriminant Analysis confirmed this pattern, with Mn and Ni showing the highest absolute discriminant coefficients, followed by Cu, while Cr contributed only marginally. Finally, HCA was performed to explore similarities among samples. The resulting dendrogram (Figure 3) strengthens the multivariate pattern, grouping all fibrous samples into a single, distinct cluster separate from the prismatic ones.

## 4 Discussion

The analysis of the tremolite samples from 11 different locations highlights significant differences in the concentrations of elements such as Ni, Mn, Cr, Cu, Zn, and other trace elements. These results are discussed in terms of their relevance to tremolite geochemistry, morphology, and its potential role in asbestos pathogenicity.

### 4.1 Nickel (Ni)

Nickel is one of the most studied and suspected toxic elements in asbestos pathogenicity. In tremolite, nickel typically occupies the crystallographic *M*(1) and *M*(3) octahedral sites within the crystal lattice (Bloise, 2023; Della Ventura et al., 1997). Our analysis showed that the Ni concentration is by far the highest (805.34 ppm) in the Bracchiello sample (Piedmont region, Italy). Once inhaled, asbestos fibers in the lungs may undergo dissolution, potentially releasing toxic elements such as Ni^2+^, which could significantly alter the biological environment and surface properties of the fibers (Perez et al., 2016).

### 4.2 Manganese (Mn)

Manganese showed considerable variability across the samples, with the highest concentration up to 1,586.17 ppm in Praborna tremolite (Aosta Valley, Northern Italy). Although manganese is not traditionally considered as toxic as other elements like nickel, it has been detected in various asbestos fibers, including amosite and crocidolite (Bloise et al., 2016). It may still contribute to the toxic effects of asbestos fibers, as its presence on the fiber surface can play a primary role in radical generation (Hernández‐Pellón & Fernández‐Olmo, 2019; Myeong et al., 2009; Riojas‐Rodríguez et al., 2010; Zoni et al., 2007). This mechanism is similar to that reported for iron (Andreozzi et al., 2017; Pacella et al., 2021) which manganese can partially substitute.

### 4.3 Chromium (Cr) and Copper (Cu)

Chromium, particularly in its trivalent form (Cr^3+^), is another element associated with asbestos pathogenicity. In tremolite, Cr^3+^ is primarily located in *M*(2) octahedral sites (Oberti et al., 2007). Our findings indicated a relatively high concentration of chromium in Campolungo sample (283.69 ppm), similar to the Cr content of other amphibole asbestos types. Unlike Fe, Cr seems not significantly contribute to the adverse radical production of asbestos. However, within lungs Cr may be leached out from the fibers through oxidative dissolution forming the genotoxic and carcinogenic hexavalent redox state (Walter et al., 2020, 2022, 2024). Copper (Cu), which also showed a higher concentration in the Campolungo sample (633.57 ppm), could further enhance the toxic potential of tremolite during prolonged fiber exposure, in agreement with the findings of Hasegawa et al. (2008). Moreover, Cu has been identified as a prooxidant and may enhance the production of free radicals, contributing to oxidative stress (Yang et al., 2023). The toxicity associated with excess copper is primarily linked to the generation of ROS, which play a role in ROS‐mediated damage at multiple biological levels (Jomova et al., 2022). The leaching of copper under physiological conditions could alter the reactivity of the fibers, affecting on how they interact with cells and tissues over time. Indeed, recent epidemiological and experimental studies have revealed significantly higher levels of copper in the sputum or lung tissues of patients with various lung diseases including lung cancer (e.g., Song et al., 2024).

### 4.4 Other Toxic Elements (Co^2+^, Pb^2+^, As^3+^)

Other toxic elements such as cobalt (Co^2+^), lead (Pb^2+^), and arsenic (As^3+^) are often identified in trace amounts in tremolite samples and are suspected contributors to the long‐term health risks associated with asbestos exposure. In our analysis, Co concentration was of 208.56 ppm in the Praborna sample, while Pb was found at 18.28 ppm in Monastero di Lanzo, and As was present in Praborna sample (66.96 ppm). These trace elements are incorporated into the fiber crystal structure and, due to their potential release during fiber leaching, could contribute to the toxicity of the tremolite (Bloise et al., 2016).

### 4.5 Mean Concentrations

**Figure 4:**
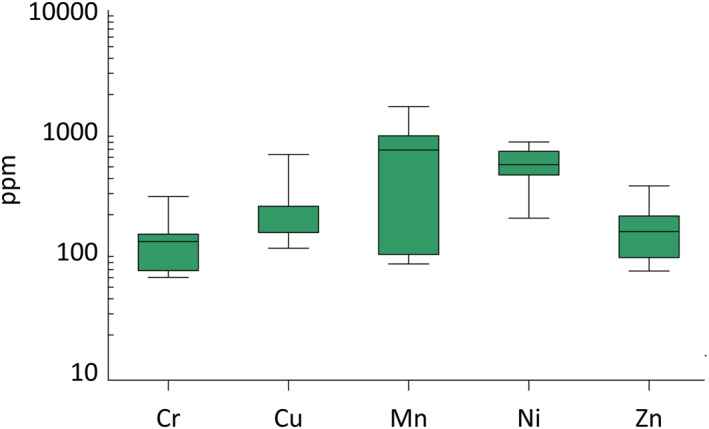
Box plots representing the concentration distributions (in ppm) of selected elements: chromium (Cr), copper (Cu), manganese (Mn), nickel (Ni), and zinc (Zn), in the tremolite asbestos samples from the 11 locations: San Severino Lucano, Val Malenco, Praborna, Monastero di Lanzo, Bracchiello, Reventino, Verrayes, Fowler, Campolungo, Iacolinei, and Caprie analyzed by Inductively Coupled Plasma Optical Emission Spectrometry.

Figure 4 shows box plots representing the concentration distributions (in ppm) of selected elements (Cr, Cu, Mn, Ni, Zn) in the analyzed tremolite asbestos samples (Table 2). The *y*‐axis is on a logarithmic scale to better visualize the variability across different elements. Elements such as Ag, As, Ba, Co, Li, Pb, Sn, and Zr were excluded from the Figure 4, as their concentrations were below the detection limit (b.d.l.) (Table 3). The average concentration of Mn (691.51 ppm) is much higher than that for the other elements. This could suggest that Mn is particularly abundant or significant in the matrix being examined, possibly due to specific geological processes. With 474.21 ppm, Ni is the second most abundant element. Copper and Chromium with 235.30 and 119.81 ppm, respectively, Cu and Cr are present in significant amounts, but still much lower than Ni and Mn. The concentration of Zn is 131.75 ppm, which places it similarly to Cu.

**Table 3:** PTEs Concentration (ppm) of Various Asbestos Tremolite

Elements (ppm)									
Locality	Fe	Co	Cr	Mn	Ni	Pb	Zn	Methods	Reference
Pena Maquieira Mine, Bragança Portugal	29,713	46	50	1,006	605	b.d.l.	28	ICP‐MS/AAS	Teixeira et al. (2010)
Bhi wara. Rajasthan, India	62,190	n.a.	1,700	1,600	700	n.a.	550	XRF	Upreti et al. (1984)
Gimigliano Calabria, Italy	24,876	22.64	170.91	1,548	308.63	4.44	28.42	ICP‐MS	Bloise et al. (2020)
Episcopia Basilicata, Italy	46,020.6	31.9	1,120	1,780	1,830	11.5	34.3	ICP‐OES	Ricchiuti et al. (2021)
Val d'Ala. Piedmont, Italy	26,258	26.92	165	1,000.123	473	0.45	17.19	ICP‐MS	Bloise et al. (2016)
Mt. Rufeno. Latium, Italy	30,818.6	b.d.l.	205	774	b.d.l.	b.d.l.	b.d.l.	EMP	Pacella et al. (2010)
Castelluccio Superiore, Basilicata Italy	28,469.2	b.d.l.	68	1,006	b.d.l.	b.d.l.	b.d.l.	EMP	Pacella et al. (2010)
San Mango, Calabria Italy	41,045.4	b.d.l.	68	619	b.d.l.	b.d.l.	b.d.l.	EMP	Pacella et al. (2010)
Ala di Stura Piedmont, Italy	33,444.4	b.d.l.	68	2012	b.d.l.	b.d.l.	b.d.l.	EMP	Pacella et al. (2010)
Montgomery County Maryland, USA	62,190	b.d.l.	b.d.l.	1,006	b.d.l.	b.d.l.	b.d.l.	EMP	Pacella et al. (2010)
Benoni Gauteng, South Africa	58,044	b.d.l.	b.d.l.	1,393	b.d.l.	b.d.l.	b.d.l.	XRD	Pooley (1975)
Val Malenco, Italy	59,179^a^	b.d.l.	81.87	906.43	532.16	b.d.l.	110.53	ICP‐OES	This work
Verrayes, Italy	29,589.5^a^	b.d.l.	137.93	568.97	715.52	b.d.l.	108.62	ICP‐OES	This work
Campolungo, Swiss	3,859.5^a^	b.d.l.	283.69	78.01	515.36	b.d.l.	347.52	ICP‐OES	This work
Iacolinei, Italy	23,400^b^	b.d.l.	120.37	898.15	333.33	b.d.l.	119.44	ICP‐OES	This work
San Severino Lucano, Italy	42,454.5^a^	b.d.l.	93.66	684.59	476.74	b.d.l.	88.04	ICP‐OES	This work
Praborna, Italy	50,173.5^a^	208.56	68.61	1,586.17	428.10	b.d.l.	195.39	ICP‐OES	This work
Fowler, USA	5,146^a^	b.d.l.	63.87	88.48	b.d.l.	b.d.l.	68.06	ICP‐OES	This work
Bracchiello, Italy	33,449^a^	b.d.l.	71.15	711.46	805.34	b.d.l.	b.d.l.	ICP‐OES	This work
Reventino, Italy	27,016.5^a^	b.d.l.	60.24	665.54	673.97	b.d.l.	68.24	ICP‐OES	This work
Monastero di Lanzo, Italy	33,449^a^	b.d.l.	255.94	1,325.41	548.45	18.28	136.74	ICP‐OES	This work
Caprie, Italy	24,443.5	b.d.l.	80.62	93.46	187.34	b.d.l.	206.74	ICP‐OES	This work
Crocidolite UICC, average value	335,123.5	5.635	13.285	755.83	21.76	4.43	20.61	ICP‐MS	Bloise et al. (2024)
Lung threshold^c^	500	0.1	0.5	3	1	0.5	30	AAS; NAA	Vanoeteren et al. (1986)
PTEs content in quartz	4.166	0.019	0.274	0.662	2.265	0.289	1.319	ICP‐MS	Götze et al. (2004)

*Note.* PTEs = potentially toxic elements. b.d.l. = below detection limit. ICP‐MS = Inductively Coupled Plasma Mass Spectrometry. ICP‐OES = Inductively Coupled Plasma Optical Emission Spectrometry. AAS = Atomic Absorption Spectroscopy. NAA = Neutron Activation Analysis. XRD = X‐ray diffraction. XRF = X‐ray fluorescence. EMP = Electron MicroProbe.

^a^ Samples previously described by Bloise (2023), with trace element concentrations determined in this study.

^b^ Samples previously described by Pacella et al. (2024), with trace element concentrations determined in this study.

^c^ Indicative baseline data for some trace elements in normal human lung tissues (Vanoeteren et al., 1986).

### 4.6 Comprehensive Elemental Assessment

**Figure 5:**
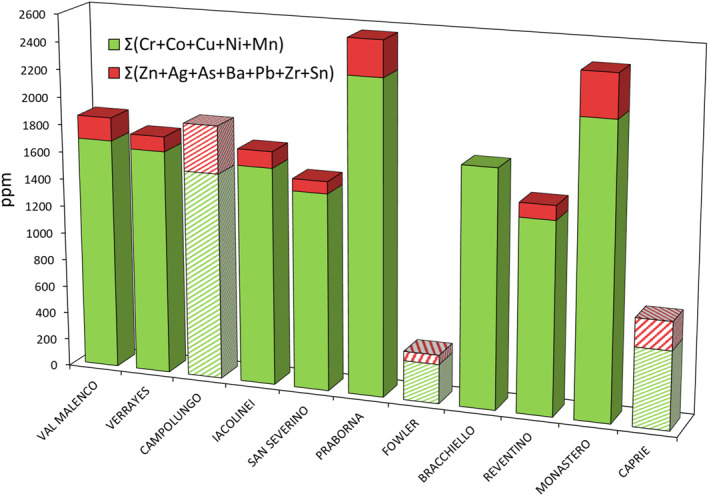
Sum of the concentrations (ppm) in the tremolite samples: San Severino Lucano, Val Malenco, Praborna, Monastero di Lanzo, Bracchiello, Reventino, Verrayes, Fowler, Campolungo, Caprie, Iacolinei. The dotted columns refer to non‐asbestos tremolite samples (Fowler, Campolungo, Caprie).

Considering the total sum of all analyzed elements (∑ Cr, Co, Ni, Cu, Mn, Zn, Ag, As, Ba, Pb, Zr, Sn), tremolite from Monastero di Lanzo exhibits the highest concentration of these elements, followed by sample from Praborna (Figure 5). The exceptionally high elemental burden in the Monastero di Lanzo tremolite likely contributes to its increased potential for long‐term toxicity. The presence of elements such as silver (Ag), arsenic (As), barium (Ba), and lead (Pb) indicates a more complex and potentially more harmful elemental profile in this sample. These elements are well‐known for their toxicity and could contribute to respiratory issues, lung cancer, and mesothelioma if inhaled. Lead (Pb) and arsenic (As), for example, are associated with significant health risks and have been linked to various types of cancers, particularly those affecting the lungs and respiratory system. Silver (Ag), although less studied in the context of asbestos toxicity, has been identified in trace amounts in some mineral samples and may also influence the reactivity of the fibers in biological fluids. Interestingly, the tremolite sample from Fowler (St. Lawrence County, New York, USA) consistently exhibited the lowest concentrations of all the elements considered. This observation suggests that the potential contribution of these elements to toxicity may be lower in the Fowler sample compared to samples from Monastero di Lanzo and Praborna.

### 4.7 Leaching and Fiber Surface Modifications

**Figure 6:**
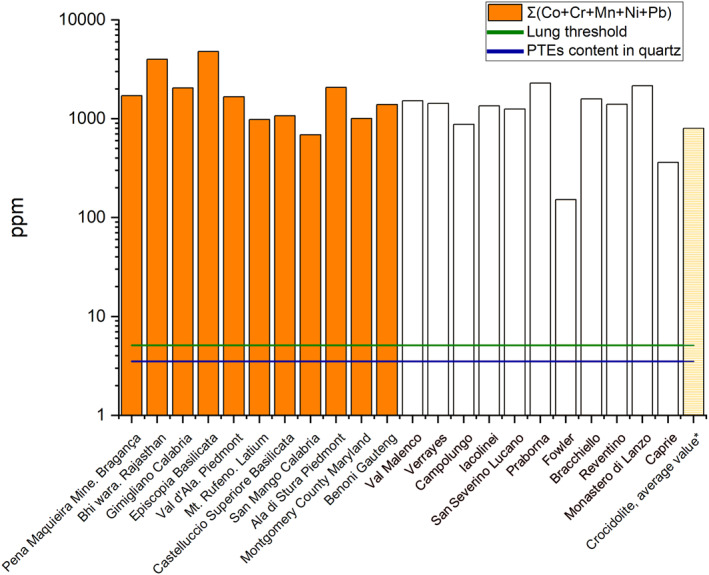
Trace element sum (ppm) in tremolite samples. Values from this study (white bars) are compared with literature data (orange bars; Bloise et al., 2016; Pacella et al., 2010; Pooley, 1975; Ricchiuti et al., 2021; Teixeira et al., 2010; Upreti et al., 1984). Average values for crocidolite from Bloise et al. (2024) are also included as striped yellow bars (see Table 3).

The dissolution of asbestos fibers in physiological fluids is a fundamental process governing their behavior in the lung and interaction with biological tissues. For tremolite, this process occurs at a slower dissolution rate compared to crocidolite and amosite, based on Si/Mg release (Pacella et al., 2023), and exhibits a clear pH dependence, with minimum rates near neutrality (Rozalen et al., 2014, 2017). A critical aspect of this mechanism is the preferential release of calcium from the M4 site, which can facilitate the breakdown of fibers into smaller, needle‐like fragments (Brantley & Chen, 1995; Diedrich et al., 2014; Schott et al., 1981). Importantly, this surface alteration, whether through acidic dissolution (e.g., in lysosomes) or oxidative leaching at physiological pH, does not fully passivate the fiber. Tremolite maintains significant surface radical activity even after prolonged leaching (Andreozzi et al., 2017), and the erosion process can lead to the intracellular release of structurally hosted PTEs (Ballirano et al., 2017). This mechanism is central to the “Trojan horse” effect, where the fiber acts as a carrier for toxic metals (Gualtieri, Gandolfi, et al., 2018; Gualtieri et al., 2019; Innes et al., 2021; Studer et al., 2010). In this context, our data reveal a higher total concentration of PTEs in tremolite compared to crocidolite (Figure 6). We hypothesize that this richer PTEs reservoir, coupled with tremolite's intermediate dissolution kinetics, could induce a distinct pathogenic pathway: potentially a more intense, metal‐driven inflammatory process compared to the chronic oxidative stress typically associated with iron‐rich fibers like crocidolite (Gualtieri, 2023).

The role of iron requires specific consideration. Although iron is a major driver of toxicity in amphibole asbestos, its pathogenicity is linked more to specific, reactive surface sites than to total bulk content (Andreozzi et al., 2017). While the bulk iron concentration in our tremolite samples is lower than in crocidolite (Table 3), it remains a significant PTEs. Therefore, the overall pathogenicity of tremolite likely arises from a synergistic effect: the cumulative release of multiple PTEs (as shown in our analysis) combined with the persistent surface reactivity of its iron sites, even after partial dissolution. This multifaceted mechanism may explain its high pathogenic potential despite its lower total iron load relative to other amphibole asbestos.

### 4.8 Geochemical Controls and Toxicological Implications of PTE Enrichment

The significant variability in PTEs concentrations among the studied tremolite samples (Figure 6; Figure S1 in Supporting Information S1) directly reflects differences in local geological environments and formation conditions. However, the concentrations of PTEs in tremolite from other locations worldwide (Bloise et al., 2016; Pacella et al., 2010; Pooley, 1975; Ricchiuti et al., 2021; Teixeira et al., 2010; Upreti et al., 1984) are consistent with the data obtained in the present study (Figure 6, Table 3). The sample from Monastero di Lanzo, for instance, shows pronounced enrichment in elements such as Ni, Cr, Co, Mn, Cu, and Zn. This specific geochemical signature likely results from a combination of factors, including interaction with a metal‐rich host rock, localized mineralization processes, and crystallization under physicochemical conditions (e.g., specific temperature, pressure, and fluid composition) that favored the partitioning of these trace elements into the amphibole structure (Della Ventura et al., 1997; Oberti et al., 2007; Tiepolo et al., 2007). In contrast, the Fowler sample exhibits the lowest PTEs levels, indicative of formation in a geochemical system lacking such enrichment mechanisms. This spatial heterogeneity underscores a fundamental principle: the toxic potential of an asbestos occurrence cannot be generalized and is intrinsically tied to its specific mineralogical and geological context. A key factor enabling this variability is the remarkable capacity of the amphibole structure to incorporate a wide range of foreign cations, a property absent in simpler framework silicates. Quartz serves as an instructive counter‐example; its structure, governed by the small ionic radius of Si^4+^ (0.42 Å), presents a highly restrictive site for cation substitution, resulting in consistently negligible PTEs concentrations (Götze et al., 2004) (Figure 6, Table 3). Tremolite, with its diverse crystallographic sites, acts as a far more efficient “sink” for PTEs (Ballirano et al., 2017).

The toxicological consequence of this geochemical enrichment is severe. Critically, the concentrations of bio‐reactive PTEs like Co, Cr, Mn, Ni, and Pb in our tremolite samples not only greatly exceed those found in inert quartz but also surpass the typical concentration ranges reported for human lung tissue (Vanoeteren et al., 1986) (Figure 6). This indicates that the inhalation of even a small mass of these fibers introduces a significant exogenous burden of toxic metals directly into the pulmonary environment, chronically exceeding local physiological baselines. Overall, the local geochemistry dictates a PTEs enrichment in tremolite that is structurally facilitated, highly variable, and of direct toxicological relevance. In this context, the fiber itself acts as a concentrated, persistent source of PTEs in the lung, representing a fundamentally distinct from that posed by minerals like quartz.

### 4.9 PTEs Content and Morphology in Tremolite

Discriminating between regulated asbestos fibers and non‐asbestos particles is a critical challenge in environmental monitoring of Naturally Occurring Asbestos (Harper et al., 2008; Van Orden et al., 2009; Wylie et al., 2022). Various techniques have been proposed to address this issue, including thermal analysis (Bloise, 2023) and morphological criteria such as crystal width (Harper et al., 2012).

Our chemical data provide further evidence for a morphological distinction. At least two out of three prismatic (non‐asbestos) tremolite samples exhibit a lower overall PTEs content compared to their fibrous (asbestos) counterparts (Figure 5), consistent with reported lower iron levels in non‐asbestos tremolite (Bloise, 2023). Statistical analysis corroborates this trend. While Mn is the only element showing a univariate significant difference between fibrous and prismatic tremolite aligning with evidence for Mn enrichment under specific conditions favoring fibrous growth (Bloise, 2023; Bloise et al., 2016) multivariate methods reveal a more comprehensive chemical signature.

MANOVA and PCA identify Mn and Ni as the primary discriminators for fibrous tremolite, whereas Cu is more associated with prismatic forms (Figure 2). Linear Discriminant Analysis confirms that fibrous tremolite possesses a coherent and distinct geochemical fingerprint. This separation is visually reinforced by cluster analysis, which groups all fibrous samples into a single cluster distinct from the prismatic ones (Figure 3).

This geochemical dichotomy reflects fundamental differences in petrogenesis. Prismatic amphiboles typically form in primary igneous or metamorphic environments under near‐equilibrium conditions, which limit trace element incorporation (Gunter et al., 2007; Langer et al., 1991). In contrast, asbestiform tremolite often crystallizes through secondary processes such as hydrothermal alteration or shearing (Addison & McConnell, 2008; Belluso et al., 2017). These dynamic, fluid‐rich environments facilitate substitution of elements such as Mg and Ca by PTEs during rapid crystal growth, resulting in the enriched and distinct chemical signature observed in the fibrous varieties.

## 5 Conclusions

This study provides a systematic quantification of PTEs in 11 tremolite asbestos, revealing a distinct elemental signature with significant implications for its toxicity and identification. Our data show that fibrous tremolite is systematically enriched in PTEs, most notably Mn (up to 691.51 ppm) and Ni (up to 474.21 ppm). Samples from localities such as Monastero di Lanzo exhibit a particularly diverse and elevated PTEs suite (Cr, Co, Ni, Cu, Zn, Ag, As, Ba, Pb, Zr, Sn), suggesting a high intrinsic toxic potential. In contrast, samples such as that from Fowler show markedly lower concentrations. Critically, multivariate statistical analysis (PCA, LDA, MANOVA) confirms that this Mn–Ni enrichment constitutes a robust geochemical fingerprint of the asbestiform habit. This signature starkly contrasts with that of prismatic/non‐asbestiform tremolite, which is characterized by low Mn–Ni and relatively higher Cu levels. This chemical dichotomy is a direct manifestation of differing formation environments: the fibrous habit crystallizes in dynamic, fluid‐rich settings that favor the incorporation of these trace elements. Therefore, our findings establish PTEs geochemistry, and specifically the Mn–Ni signature, as a critical diagnostic tool. It should be integrated with established morphological and crystallographic methods to improve the reliable discrimination of regulated asbestos tremolite from its non‐asbestiform counterparts a persistent challenge in environmental monitoring.

From a toxicological perspective, the significant reservoir of bio‐accessible PTEs (notably Ni, Cr, Co, Pb) hosted within the tremolite structure provides a direct mechanistic link to its pathogenicity. These elements, likely released via fiber dissolution in the lung, can contribute to both acute and chronic toxicity, potentially explaining the high mesothelioma risk associated with tremolite exposure. In conclusion, this work advances the understanding of tremolite asbestos by directly linking its hazardous nature to a definitive geochemical composition. The quantitative PTE profiles presented here are essential for interpreting in vitro toxicity assays and for guiding future research into the specific mechanisms of asbestos‐induced diseases.

## Ethics Statement

No specific ethics approval was required for this study as data analysis was based on publicly available data.

## Conflict of Interest

The authors declare no conflicts of interest relevant to this study.

## Supporting information

Supporting Information S1

## Data Availability

The data supporting this study are available in Bloise et al. (2026). Moreover, part of the data is available in Bloise (2023). Statistical analyses were performed using PAST software, version 4.17 https://www.nhm.uio.no/english/research/resources/past/. Data analysis and graphing were performed using OriginPro 2019 (OriginLab Corporation, Northampton, MA, USA).
