# Supplementary material for: Mineralogical and Geochemical Fingerprinting of Potentially Toxic Elements (PTEs) in Asbestos and Non‐Asbestos Tremolite: Implications for Human Health
Source: Geohealth. 2026 May 20;10(5):e2026GH001853. doi: 10.1029/2026GH001853 (PMC13239513; doi:10.1029/2026GH001853)
Supplement: Supplementary file 1 — Supporting Information S1 [file GH2-10-e2026GH001853-s001.pdf]

GeoHealth

Supporting Information for

Mineralogical and Geochemical Fingerprinting of Potentially Toxic Elements (PTEs) in asbestos and non-asbestos tremolite: Implications for Human Health

A. Bloise[1,2,3*], I. Fuoco[1, *], G. Vespasiano[1], E. Giorno[4], A. Pacella[5], S. Filicetti[1], M.F. La Russa[1], D. Pereira[3,6], C. Piersante[1], C. Apollaro[1]

[1]Department of Biology, Ecology and Earth Sciences, University of Calabria, I-87036 Rende, CS, Italy

[2]University Museum System – SiMU, University of Calabria, I-87036 Rende, CS, Italy

[3]Research Group CHARROCK, University of Salamanca, 37008 Salamanca, Spain

[4]Department of Chemistry and Chemical Technology Department, University of Calabria, I-87036 Rende, CS, Italy

[5]Department of Earth Sciences, Sapienza University of Rome, Piazzale Aldo Moro 5, I-00185, Rome, Italy

[6]Geology Department, Science Faculty, University of Salamanca, Plaza Merced s/n, Salamanca 37008, Spain

Contents of this file

Text S1 to S2
Figures S1 to S2
Table S1

Text S1.

Supplementary Figure 1 shows the concentrations of selected trace elements measured in tremolite samples from eleven localities (Val Malenco, Verrayes, Campolungo, Caprie, Iacolinei, San Severino Lucano, Praborna, Fowler, Bracchiello, Reventino, and Monastero di Lanzo). The analysed elements include silver (Ag), arsenic (As), barium (Ba), cobalt (Co), chromium (Cr), copper (Cu), lithium (Li), manganese (Mn), nickel (Ni), lead (Pb), tin (Sn), zinc (Zn) and zirconium (Zr). All concentrations were determined by inductively coupled plasma–optical emission spectrometry (ICP-OES) after acid digestion of the samples, following the same analytical protocol described in the Materials and Methods section of the main manuscript. Because of the wide range of element concentrations across the dataset, values are plotted on a logarithmic scale on the Y-axis to facilitate the visualization of both low- and high-abundance elements within the same graph.

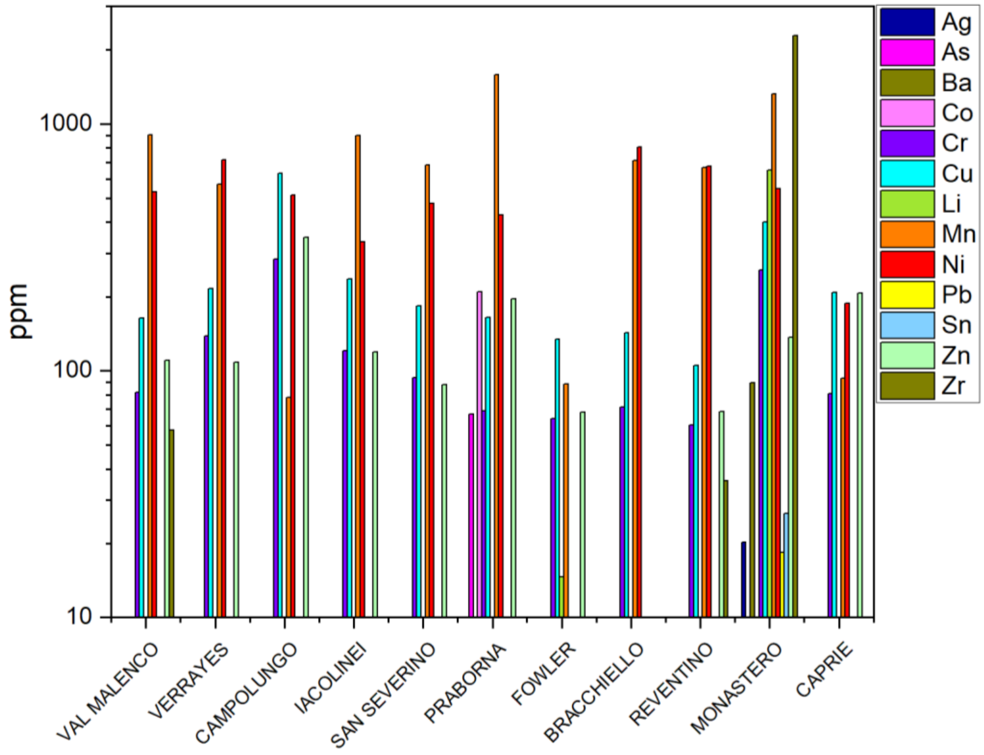

Figure S1. Trace element concentrations (ppm) in tremolite samples determined by ICP-OES

Text S2.

Supplementary Figure S2 shows zinc (Zn) concentrations measured in tremolite samples from eleven localities (San Severino Lucano, Val Malenco, Praborna, Monastero di Lanzo, Bracchiello, Reventino, Verrayes, Fowler, Campolungo, Caprie, and Iacolinei). Zinc concentrations were determined by inductively coupled plasma–optical emission spectrometry (ICP-OES) following acid digestion of the samples, using the same analytical protocol described in the Materials and Methods section of the main manuscript. In the figure, dotted and dark columns identify non-asbestos tremolite samples.

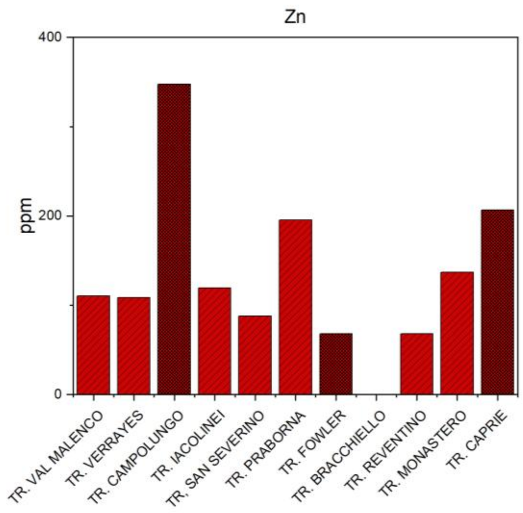

Figure S2. Zinc (Zn) concentrations (ppm) in tremolite samples determined by ICP-OES. The dotted and dark columns refer to non-asbestos tremolite samples.

Table S1. Average (at least 5-point analyses) major and minor element concentrations (wt%) in the investigated tremolite samples, as detected by EPMA/EDS. Standard deviations in brackets. From Bloise, 2023.

Oxides	Tremolite San Severino Lucano	Tremolite Val Malenco	Tremolite Maryland	Tremolite Praborna	Tremolite Monastero di Lanzo	Tremolite Bracchiello	Tremolite Reventino	Tremolite Verrayes	Tremolite Ala	Tremolite Caprie	Tremolite Fowler	Tremolite Campolungo
**SiO_2**	58.6 (6)	58.3 (9)	58.4 (6)	57.5 (15)	58.7 (2)	59.5 (1)	58.9 (4)	58.3 (1)	57.1 (2)	58.2 (09)	58.4 (14)	58.1 (16)
**MgO**	24.1 (15)	22.7 (13)	22.7 (4)	23.0 (13)	23.2 (1)	24.0 (2)	25.2 (1)	22.3 (19)	24.6 (2)	23.7 (07)	23.4 (24)	24.0 (11)
**CaO**	13.8 (11)	14.4 (18)	14.6 (3)	14.3 (16)	15.5 (2)	13.7 (4)	13.7 (1)	15.5 (27)	15.2 (6)	14.1 (14)	15.8 (41)	15.9 (17)
**FeO**	3.3 (9)	4.6 (5)	4.3 (4)	3.9 (14)	2.6 (2)	2.6 (1)	2.1 (3)	2.3 (2)	1.9 (2)	1.9 (3)	0.4 (02)	0.3 (1)
**Na_2O**	0.2 (1)	n.d.	n.d.	0.6 (2)	n.d.	0.1 (2)	n.d.	0.9 (1)	0.8 (6)	1.7 (2)	0.9 (04)	0.8 (4)
**Al_2O_3**	0.1 (1)	n.d.	n.d.	0.8 (1)	n.d.	n.d.	0.2 (4)	0.8(1)	0.6 (1)	0.4 (1)	1.1 (01)	0.9 (5)
**Sum**	100.0	100.0	100.0	100.0	100.0	100.0	100.0	100.0	100.0	100.0	100.0	100.0
CATIONS calculated on the basis of 23 oxygens												
**Si**	7.82	7.90	7.91	7.79	7.91	7.94	7.83	7.88	7.70	7.83	7.83	7.86
**$Mg/(Mg+Fe^{2+})$**	0.99	0.90	0.90	0.91	0.94	0.98	1.00	0.95	0.96	0.96	0.99	0.99
